# Dysphagia Secondary to Cervical Hyperostosis in Diffuse Idiopathic Skeletal Hyperostosis: A Case Report

**DOI:** 10.7759/cureus.109420

**Published:** 2026-05-22

**Authors:** Ali Mohamed, Harvey Lai, Nikkie Verhagen, Suranga Singhapathirana, Saira Mushtaq, Kamala Jegu, Mohamed K Mansour

**Affiliations:** 1 General and Acute Medicine, Royal Hobart Hospital, Hobart, AUS; 2 Medicine, Teaching Hospital Batticaloa, Columbo, LKA; 3 Internal Medicine, Rochester Regional Health/Unity Hospital, Rochester, USA

**Keywords:** diffuse idiopathic skeletal hyperostosis (dish), dysphagia, dysphagia requiring surgical relief, forestier disease, rare cause of dysphagia

## Abstract

Dysphagia in older adults is commonly attributed to intrinsic oesophageal pathology. Extrinsic mechanical causes of dysphagia due to uncommon conditions are often overlooked. Diffuse idiopathic skeletal hyperostosis (DISH) is a non-inflammatory disorder characterised by ossification of spinal ligaments and may cause dysphagia when the cervical spine is involved.

We describe a 79-year-old man presenting with progressive dysphagia, weight loss, and aspiration secondary to extrinsic compression by cervical osteophytes in the context of DISH. The patient underwent multilevel anterior cervical discectomy and fusion with osteophytectomy, followed by swallow rehabilitation, resulting in resolution of his symptoms.

This case emphasises the importance of considering cervical DISH in the differential diagnosis of elderly patients presenting with unexplained dysphagia. It also highlights the value of imaging in establishing the diagnosis of this condition.

## Introduction

Dysphagia, or difficulty swallowing, is a common clinical complaint among older adults and often presents a diagnostic challenge due to its broad differential diagnosis. While intrinsic oesophageal or neuromuscular disorders are frequently considered, extrinsic mechanical compression is a less commonly recognised aetiology. Among these causes, diffuse idiopathic skeletal hyperostosis (DISH), also known as Forestier’s disease, represents an underdiagnosed source of oropharyngeal and oesophageal symptoms. DISH is a non-inflammatory disorder characterised by calcification and ossification of the anterior longitudinal ligament (ALL) and other entheses. It most commonly affects the thoracic spine but may have significant clinical consequences when the cervical region is involved [1-3].

Cervical DISH may remain asymptomatic or present insidiously with dysphagia, globus sensation, odynophagia, dysphonia, stridor, and, in severe cases, aspiration pneumonia or airway obstruction [4-6]. Dysphagia typically results from direct oesophageal compression by prominent anterior osteophytes, particularly at fixed anatomical levels such as the cricoid region (C5-C6), or from associated inflammatory and spastic mechanisms [3,7].

Although the radiological hallmarks are well defined, including flowing anterior ossification across at least four contiguous vertebrae without significant disc degeneration or facet joint ankylosis, the diagnosis is frequently delayed or overlooked [3,8]. This case highlights a rare but increasingly recognised presentation of DISH with progressive dysphagia secondary to anterior cervical osteophytes. It underscores the importance of considering DISH in elderly patients presenting with unexplained swallowing difficulties and highlights the role of targeted cervical spine imaging as an adjunct to standard oesophageal investigations in establishing the diagnosis.

## Case presentation

A 79-year-old man presented to the Royal Hobart Hospital with progressive dysphagia, significant weight loss, and a choking episode during meals, as well as limited ability to turn and flex his neck. His symptoms had worsened over the preceding three to six months and included hoarseness, coughing during swallowing, regurgitation, and reduced oral intake. His medical history was significant for total gastrectomy with Roux-en-Y reconstruction in 2015 for gastric adenocarcinoma (T3N0M0). His most recent surveillance endoscopy in 2021 showed no evidence of recurrence. 

On examination, the patient was alert and oriented with dysphonia but no stridor or respiratory distress. Neck examination demonstrated reduced cervical range of motion, particularly in flexion and rotation. There were no palpable cervical masses or lymphadenopathy.

Initial investigations included gastroscopy, which demonstrated altered gastric anatomy secondary to prior surgery but no mucosal abnormalities or structural cause for his dysphagia. Biopsies from the proximal oesophagus revealed only mild, non-specific reactive changes, with no evidence of malignancy. Given progressive dysphagia, weight loss, choking episodes, dysphonia, and absence of an intrinsic oesophageal cause on gastroscopy, a videofluoroscopic swallow study (VFSS) was performed to assess swallow safety, aspiration risk, and possible pharyngeal-level or extrinsic mechanical obstruction.

**Figure 1:**
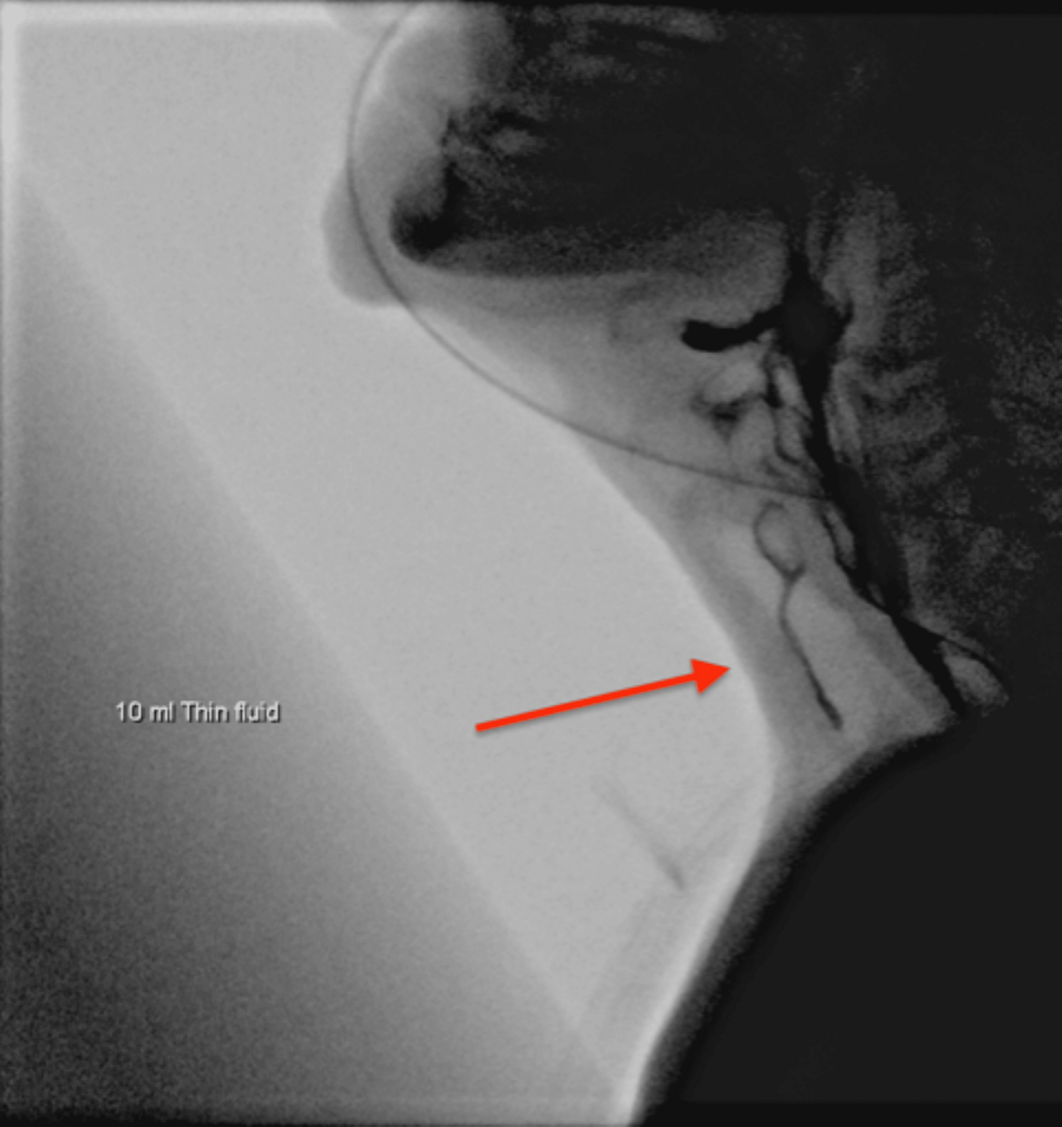
VFSS demonstrating silent aspiration (arrow) and external compression of the oesophagus by a large anterior osteophyte Original X-ray image from Royal Hobart Hospital, Australia, 2025, reproduced with permission from Royal Hobart Hospital Radiology Department VFSS: Videofluoroscopic swallow study

The VFSS confirmed severe pharyngeal dysphagia with aspiration of all consistencies and ineffective bolus clearance (Figure 1). The barium swallow demonstrated mechanical obstruction with delayed swallow initiation and significant pharyngeal residue associated with silent aspiration. The severity of dysphagia was consistent with severe impairment according to the Dysphagia Outcome and Severity Scale.

A contrast-enhanced computed tomography (CT) scan of the neck was performed to exclude an enhancing mass or alternative structural pathology, given the patient’s weight loss, dysphonia, and prior history of gastric adenocarcinoma.

**Figure 2:**
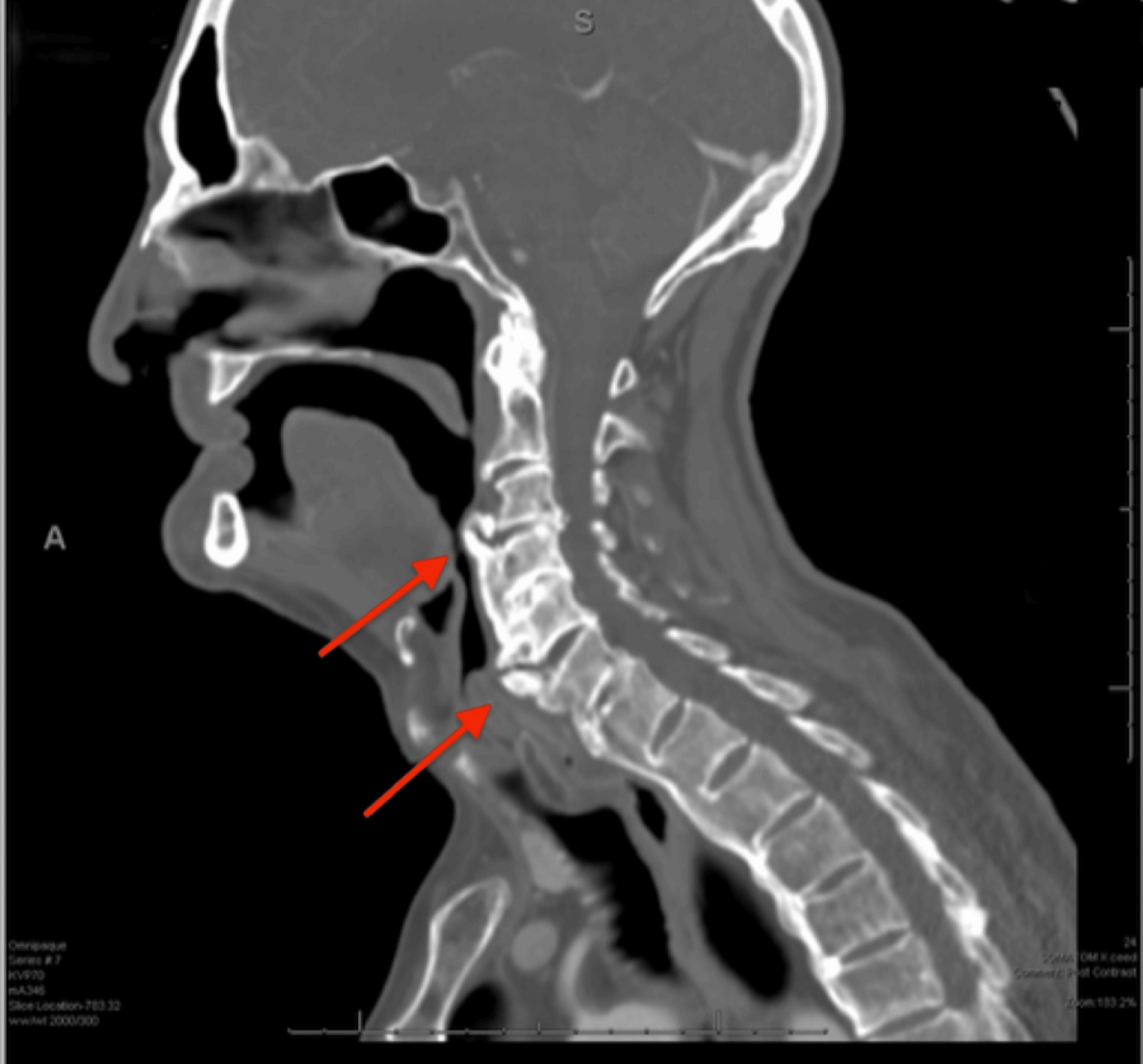
Preoperative contrast-enhanced CT of the neck demonstrating a markedly prominent C5/C6 osteophyte compressing the left pyriform fossa. No abnormal lymph nodes were identified. Original CT image from Royal Hobart Hospital, Australia, 2025, reproduced with permission from Royal Hobart Hospital Radiology Department

**Figure 3:**
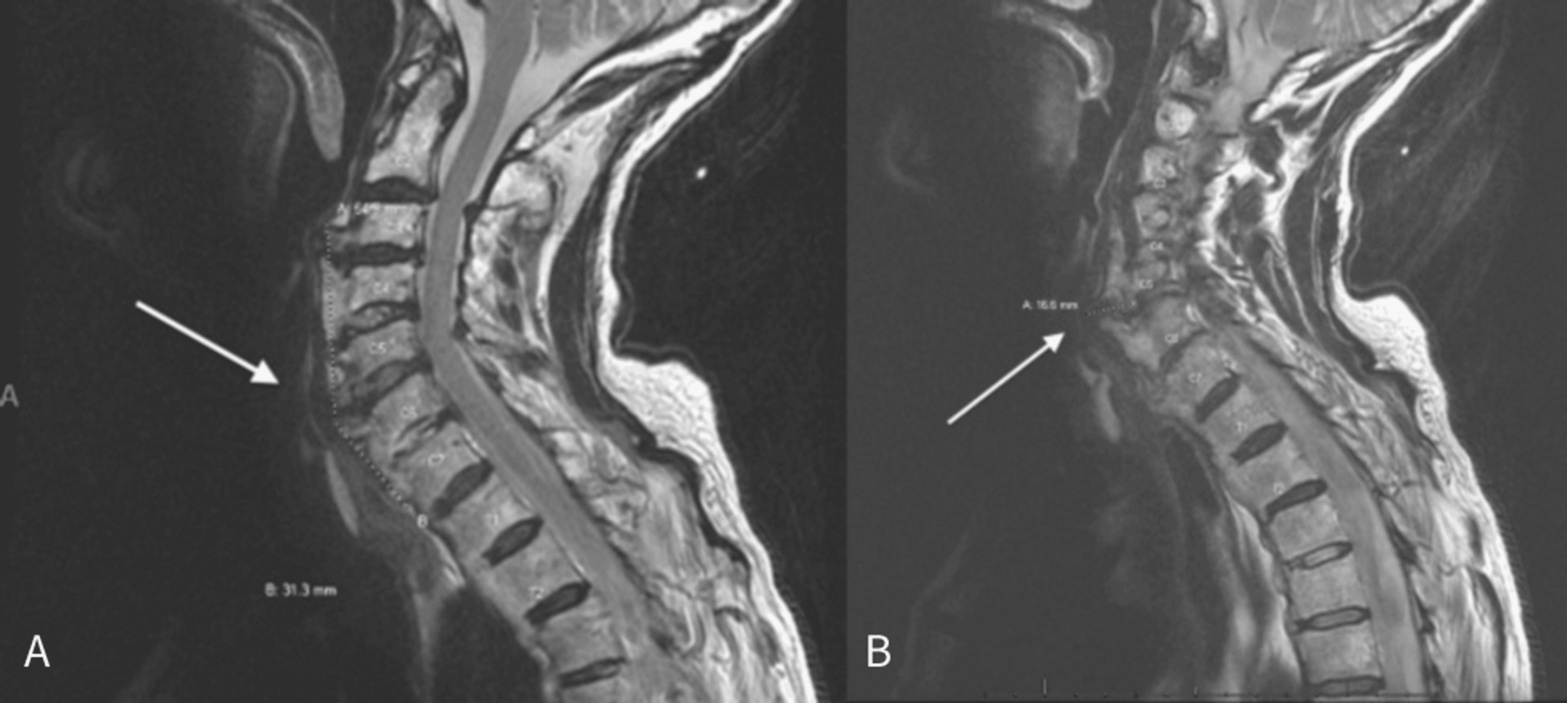
(A and B) Preoperative T2-weighted sagittal MRI of the spine demonstrating bulky, flowing osteophytes along the anterior aspect of the cervical and upper thoracic spine (white arrows). Original MRI image from Royal Hobart Hospital, Australia, 2025, reproduced with permission from Royal Hobart Hospital Radiology Department

The scan demonstrated no enhancing masses but revealed a markedly prominent anterior osteophyte at the C5/C6 level, causing severe compression of the left pyriform fossa (Figure 2). This finding was further confirmed by magnetic resonance imaging (MRI) of the cervical spine, which showed extensive ossification of the ALL with large flowing osteophytes consistent with DISH. The largest osteophyte measured 17 mm in diameter at C5/C6 and extended over a span of 85 mm from C3 to C7. These osteophytes were noted to indent both the oropharynx and laryngopharynx (Figure 3).

Due to ongoing weight loss and the risk of aspiration, the patient was made nil by mouth and commenced on nasogastric tube (NGT) feeding for nutritional support. Multidisciplinary discussions among the ENT, neurosurgery, speech pathology, and gastroenterology teams concluded that the dysphagia was predominantly mechanical in nature and attributable to cervical osteophytes in the context of DISH.

**Figure 4:**
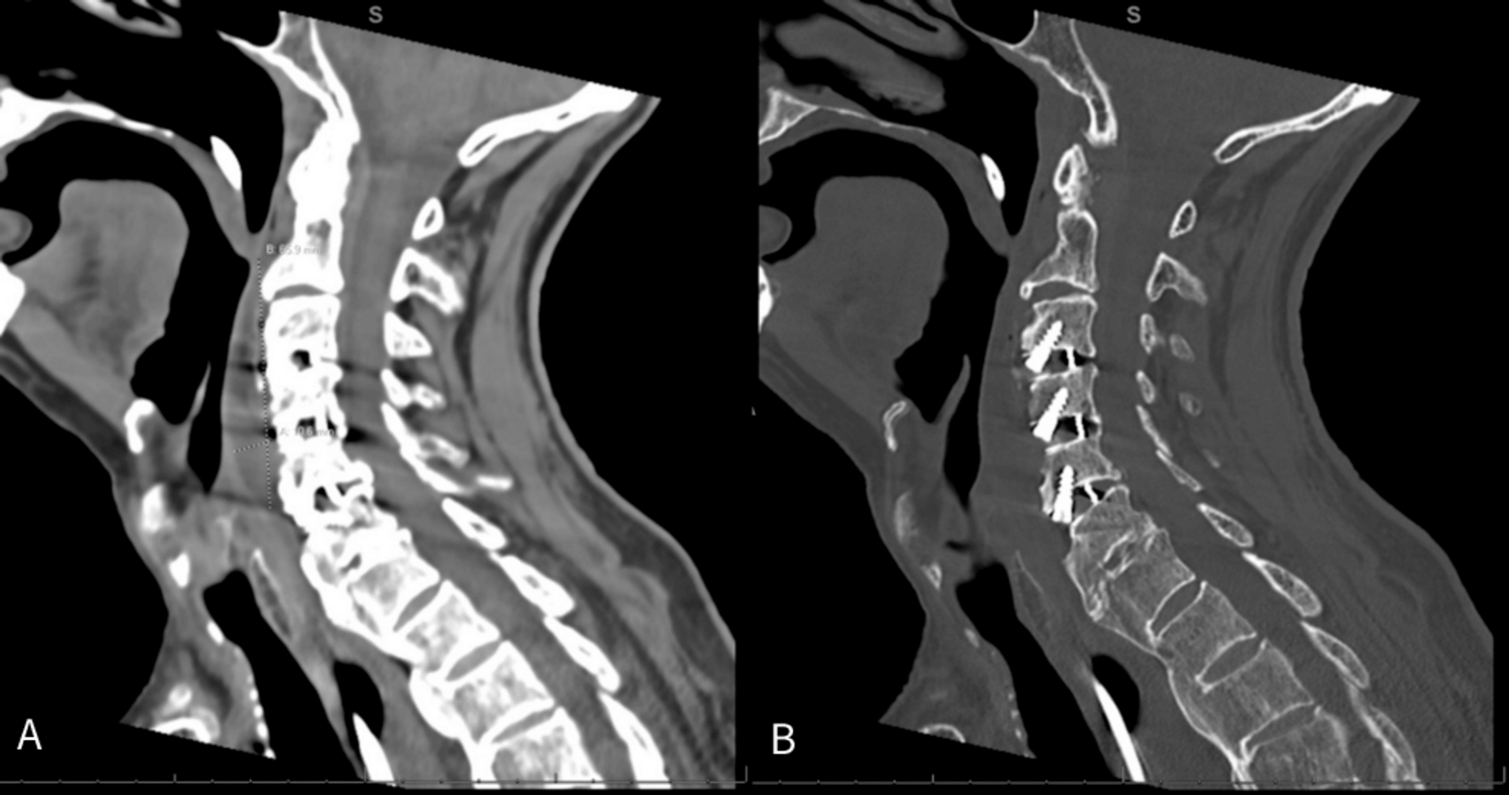
(A and B) Postoperative sagittal CT of the cervical spine demonstrating restoration of normal cervical vertebral alignment and anatomy. Original CT image from Royal Hobart Hospital, Australia, 2025, reproduced with permission from Royal Hobart Hospital Radiology Department

Following preoperative workup and nutritional optimisation, the patient underwent anterior cervical discectomy and fusion (ACDF) at C3/4, C4/5, and C5/6. Intraoperatively, the osteophytes were drilled and removed, and intervertebral cages with appropriate screw fixation were inserted. A postoperative CT scan confirmed satisfactory hardware positioning with expected postoperative changes, including a small prevertebral haematoma that did not compromise the airway (Figure 4).

**Figure 5:**
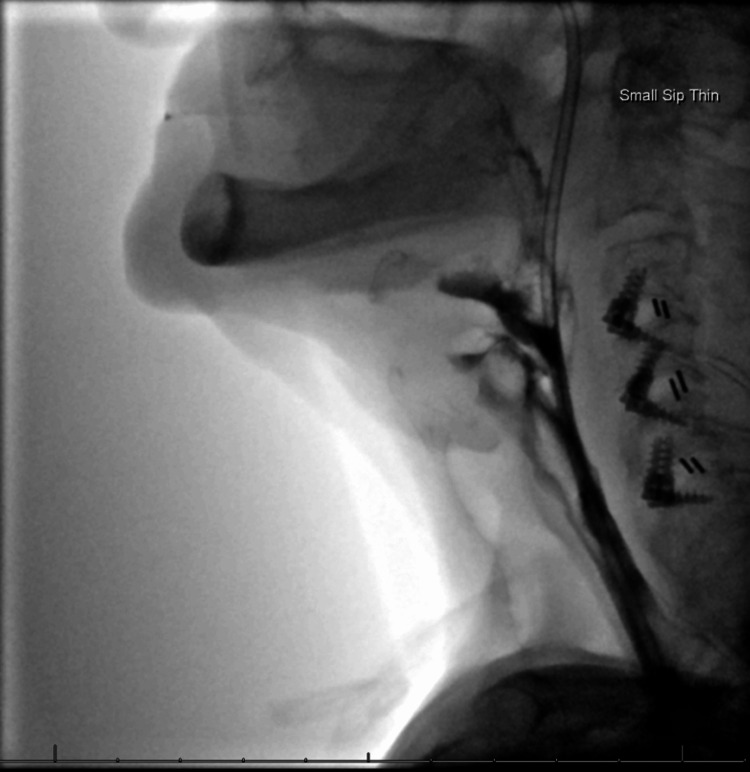
VFSS performed three days postoperatively demonstrating contrast passage through the pharynx into the oesophagus without evidence of aspiration or airway penetration on this swallow sequence. Original X-ray image from Royal Hobart Hospital, Australia, 2025, reproduced with permission from Royal Hobart Hospital Radiology Department VFSS: Videofluoroscopic swallow study

Postoperatively, he remained on NGT feeding and was reviewed by speech pathology. A follow-up VFSS study was performed (Figure 5). A tailored swallow rehabilitation program was implemented. At the time of writing, the patient was clinically stable, without evidence of airway compromise, and was discharged from the hospital with a multidisciplinary care plan in place.

## Discussion

DISH is a non-inflammatory systemic condition that predominantly affects older adults and is often underrecognised. The hallmark of DISH is calcification and ossification of ligaments and entheses, most notably along the anterior aspect of the spine, leading to the formation of prominent osteophytes [9].

Recent epidemiological analyses suggest that the prevalence of DISH increases with age, with some studies estimating that more than 10% of individuals over 70 years are affected [10]. The demographic distribution appears to favour men, as demonstrated in a study conducted in Finland in which 10.1% of men and 6.8% of women above 70 years exhibited radiographic evidence of the disease [11].

The aetiology of DISH remains unclear. However, several risk factors have been identified, including metabolic conditions such as obesity, diabetes, and genetic predisposition [12]. Some investigators have proposed that abnormal osteoblastic activity at the enthesis, possibly resulting from reduced inhibitory regulatory factors, may contribute to osteophyte formation. Environmental exposures, including vitamin A and retinoid intake, have also been considered possible contributors, although their precise roles remain incompletely understood. Rather than a single causative pathway, current evidence supports DISH as a disorder arising from the interplay of metabolic, mechanical, genetic, and environmental influences. This perspective highlights the complexity of its pathogenesis and underscores the need for further research addressing these intersecting domains.

In this case, the cervical vertebral bodies were fused by bridging anterior osteophytes resulting from ossification of the ALL. DISH most commonly affects the thoracic spine; however, cervical involvement is more likely to produce clinically significant symptoms. The slowly progressive nature of DISH often results in asymptomatic or mildly symptomatic disease. However, depending on location and involvement of adjacent structures, a range of clinical consequences may occur. Prominent cervical osteophytes may compress the oesophagus and cause dysphagia, as demonstrated in this case, which is among the more common symptomatic cervical manifestations and has been increasingly recognised in recent years [13]. In this patient, bulky flowing ossification of the ALL involved the cervical and upper thoracic spine. The largest components of calcification were present at C3/C4, indenting the oropharynx, and at C5/C6, indenting the laryngopharynx.

The clinical manifestation of dysphagia in DISH is widely recognised as multifactorial, with symptoms often more pronounced with solid foods [14]. It may be accompanied by a foreign body sensation and, in rare cases, may progress to complete oesophageal obstruction [15], underscoring the spectrum of clinical presentations. Smaller osteophytes located at anatomical fixation points, such as the cricoid cartilage, have been implicated in symptom exacerbation through mechanisms involving pain and muscle spasm [16]. These mechanisms are thought to disrupt the swallowing phase, impairing coordinated transfer of the bolus from the oral cavity through the pharynx and upper oesophageal sphincter into the oesophagus [17]. 

Although ankylosing spondylitis can share some imaging features with DISH, it typically affects younger patients and is characterised by inflammatory sacroiliitis and syndesmophyte formation, in contrast to the flowing anterior ligamentous ossification and preserved disc spaces seen in DISH [18].

The presentation of dysphagia and airway obstruction in DISH remains uncommon despite increased recognition of the condition in clinical practice. Cervical DISH therefore represents an uncommon but underrecognised cause of dysphagia in older adults, accounting for only a small proportion of dysphagia presentations despite the relatively high prevalence of DISH in this age group. A recent meta-analysis conducted by Harlianto et al. identified 419 published cases over an 11-year period [19], highlighting its rarity and potential challenges in diagnosis. It is important to note that the presence of DISH does not uniformly translate into symptomatic disease. As observed by Nishimura et al., the severity of dysphagia in cervical spine DISH is likely influenced by osteophyte thickness, cervical spine mobility, and cranio-cervical positioning [14]. These findings highlight the complex interplay of anatomical variables in symptom expression.

It has been hypothesised that slowly growing cervical bony protuberances are generally well tolerated by many individuals, but this tolerance may occur at the expense of reduced compensatory reserve. When a trigger event, such as aspiration, regurgitation, sleep apnoea, upper respiratory tract infection, or minor cervical trauma, occurs, it may induce soft tissue swelling around an already mechanically compromised oesophagus or trachea. This sudden change may overwhelm compensatory mechanisms and precipitate acute dysphagia and/or airway obstruction [20]. Dysphagia in DISH often improves with neck flexion [14]. Patients may also experience odynophagia, saliva stagnation, dysphonia, and dyspnoea [21].

Cervical involvement in DISH can cause complications beyond dysphagia, including hoarseness, stridor, aspiration pneumonia, atlantoaxial subluxation, and pseudoarthrosis [13,22-24]. Less commonly, patients may experience airway compromise, sleep apnoea, and thoracic outlet syndrome [25,26]. Aspiration has been reported more frequently in patients with osteophytes larger than 10 mm [27], as observed in this case. When larger segments of the cervical spine are affected, both oesophageal and laryngeal symptoms may develop [22]. Large osteophytes can exert significant mechanical pressure on adjacent soft tissues, making procedures requiring neck hyperflexion, such as endotracheal intubation, more challenging [28-30].

In the present case, a comprehensive diagnostic approach was employed, including X-ray, CT, MRI, VFSS, barium swallow, and gastroscopy. The diagnosis of DISH is primarily radiographic and is often first suggested on plain lateral spine radiographs. Plain radiographs typically demonstrate flowing calcification and ossification along the anterior surface of the vertebrae, spanning at least four contiguous vertebrae, in the absence of spondyloarthropathy or significant degenerative spondylosis. The hyperostosis often presents as a “drooping drop,” which progressively enlarges to form spurs until bridging adjacent vertebral bodies [31].

**Figure 6:**
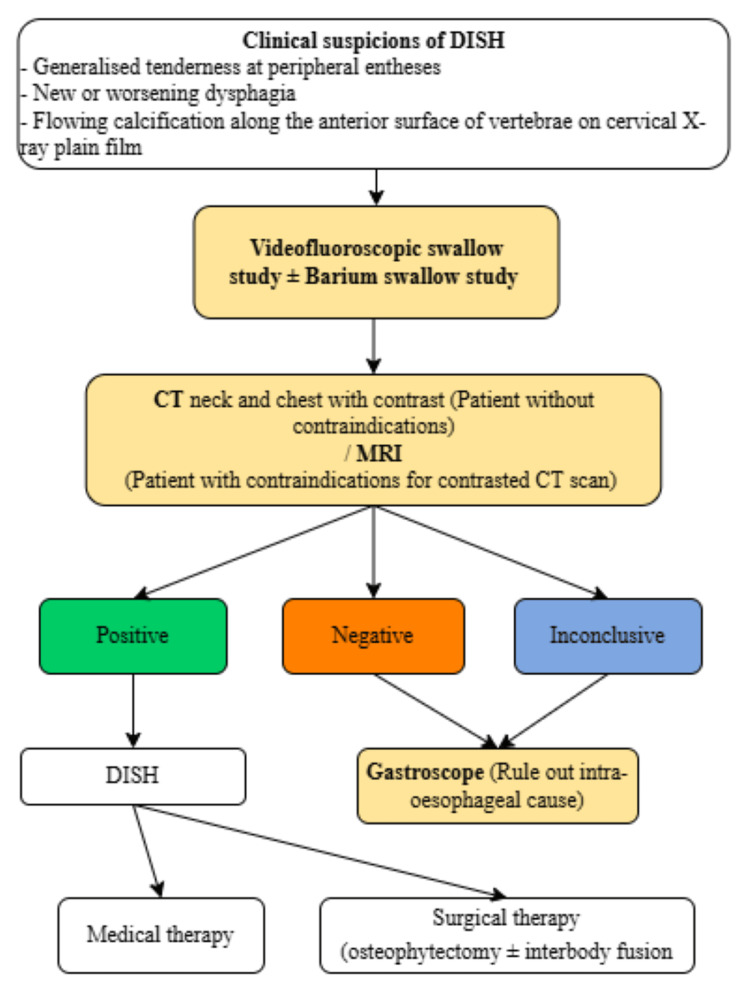
Proposed diagnostic approach. Created by authors in drawio (https://www.drawio.com/) DISH: Diffuse idiopathic skeletal hyperostosis

CT and MRI are valuable for assessing the extent of hyperostosis and its relationship to the oesophagus, as well as for identifying complications such as spinal stenosis and compressive myelomalacia [32,33]. Although DISH itself can usually be adequately demonstrated on non-contrast CT, contrast-enhanced CT may be useful when alternative pathology, such as malignancy, lymphadenopathy, or pharyngeal structural abnormalities, is part of the differential diagnosis, as in this case. Additionally, both CT and MRI play a role in risk stratification for dysphagia prior to surgical intervention [2]. To clarify the diagnostic approach and assist clinical decision-making, the authors have created a flow diagram proposing an approach to diagnosing dysphagia secondary to DISH (Figure 6).

For patients with degenerative cervical spine disease, dysphagia, and suspected osteophytes, it is essential to perform a barium swallow or VFSS. These studies help determine whether osteophytes are causing oesophageal obstruction [34-36]. Barium swallow typically demonstrates a narrowed segment due to osteophyte compression, as observed in this case, and may be repeated for follow-up evaluation. To exclude intrinsic oesophageal pathology, such as oesophagitis or tumours, endoscopy is also necessary. However, endoscopy should be performed with caution in patients with cervical osteophytes due to the increased risk of oesophageal perforation [37].

Initial management of DISH generally prioritises conservative measures. These include non-steroidal anti-inflammatory drugs, dietary modification such as transition to softer foods, corticosteroid injections, and muscle relaxants [38-40]. Physiotherapy also plays an important role in maintaining mobility and relieving pain [41]. Given the association of DISH with metabolic changes and visceral obesity, patient education regarding physical activity and dietary modification, particularly reduction in saturated fats and refined carbohydrates, is important [42,43].

Surgical intervention is usually reserved for severe cases, particularly in patients with progressive airway obstruction or dysphagia that does not respond to conservative therapy [44]. The primary surgical approach involves direct anterior resection of osteophytes to relieve mechanical compression and restore functional reserve [45]. Most patients experience improvement in dysphagia following cervical osteophyte resection [33,46]. In cases with concurrent cervical spine instability or cord compression, interbody fusion may be indicated [36]. Interbody fusion can enhance stability and may theoretically reduce osteophyte recurrence. The use of plates and screws during spinal fusion may also act as a barrier to limit local bone regrowth, thereby potentially lowering recurrence risk. However, the decision to perform fusion should be individualised based on the underlying pathology.

Although recurrence of osteophytes after surgical excision is uncommon, follow-up studies have documented cases of regrowth that sometimes require repeat surgical intervention [21,47-51]. Evidence suggests that recurrent cervical osteophytes may develop after resection, with an average regrowth rate of approximately 1-2 mm per year [44,48]. Consequently, interbody fusion is sometimes considered to reduce this risk [48,49,52]. Despite these strategies, the comparative efficacy of interbody fusion versus osteophytectomy alone remains uncertain, as direct comparative studies are lacking.

Given the potential for delayed recurrence, extended long-term follow-up is recommended for patients who have undergone surgery. To further reduce the likelihood of postoperative ossification, prophylactic strategies such as indomethacin therapy or targeted radiotherapy have been described in the literature [36].

During surgical exposure of large osteophytes, there is a significant risk of structural injury, as the oesophagus can be difficult to mobilise and may adhere to surrounding cervical structures due to local inflammation. Surgery has been associated with complications including oesophageal injury, recurrent laryngeal nerve injury, stroke, Horner’s syndrome, and cervical instability [53]. Some authors advocate for a more proactive surgical approach in patients with cervical osteophytes causing dysphagia or dyspnoea, given the risk of perforation and acute airway compromise [34,54]. However, this must be balanced against procedural risks. Consequently, a more conservative strategy is generally adopted, reserving surgery for patients with persistent or severe symptoms that do not respond to non-operative management [55,56].

In the present case, the largest lesion segment was identified at C5/C6, a region associated with high mechanical stress and significant anterior soft tissue compression. Therefore, ACDF was performed.

## Conclusions

DISH is a relatively common yet frequently underrecognised condition, often remaining undiagnosed due to its asymptomatic nature. This case highlights the importance of including DISH in the differential diagnosis of dysphagia, particularly in older adults with prominent anterior cervical osteophytes on imaging and unremarkable gastroscopic findings. As the pathogenesis of DISH remains incompletely understood, diagnosis continues to rely primarily on radiographic evaluation, with plain cervical spine radiographs serving as an initial tool and barium swallow studies or advanced imaging such as CT and MRI helping to define the extent of disease and associated complications.

Management should be individualised based on symptom severity and functional impairment. Conservative approaches, including dietary modification and symptomatic treatment, are appropriate for mild cases, while surgical intervention (surgical osteophytectomy with or without interbody fusion) may be warranted in patients with severe or progressive symptoms, especially when significant mechanical compression or aspiration risk is present. The lack of robust, evidence-based guidelines highlights the need for multidisciplinary care and ongoing follow-up. This case contributes to the existing literature and reinforces the need for greater clinical awareness of cervical DISH as an important and potentially treatable cause of dysphagia in older adults.
