# Correction: Dysphagia Secondary to Cervical Hyperostosis in Diffuse Idiopathic Skeletal Hyperostosis: A Case Report

**DOI:** 10.7759/cureus.c450

**Published:** 2026-06-12

**Authors:** Ali Mohamed, Harvey Lai, Nikkie Verhagen, Suranga Singhapathirana, Saira Mushtaq, Kamala Jegu, Mohamed K Mansour

**Affiliations:** 1 General and Acute Medicine, Royal Hobart Hospital, Hobart, AUS; 2 Medicine, Teaching Hospital Batticaloa, Columbo, LKA; 3 Internal Medicine, Rochester Regional Health/Unity Hospital, Rochester, USA

The author list has been corrected due to a technical error displaying the incorrect name for author Suranga Singhapathirana.

